# Recreational sports in prison: inmates' perspectives on coaching effectiveness

**DOI:** 10.3389/fspor.2024.1488600

**Published:** 2024-12-04

**Authors:** Milan Dransmann, Lara Lesch, Bernd Gröben, Pamela Wicker

**Affiliations:** Department of Sports Science, Bielefeld University, Bielefeld, Germany

**Keywords:** offender, rehabilitation, coach, interviews, content analysis, knowledge, outcomes, context

## Abstract

**Introduction:**

This study examines inmates' perspectives on the effectiveness of sports coaches in prison. According to the integrative definition of effective coaching, coaches require professional, interpersonal, and intrapersonal knowledge to enhance athletes' outcomes within a specific context.

**Methods:**

Five male inmates of a German prison were interviewed after they participated in sports programs. The data were analyzed using directed content analysis.

**Results:**

The inmates recognized professional knowledge in coaches who demonstrated clear training structures and spoke in an educated manner. Interpersonal knowledge was highly valued, with inmates expressing a preference for coaches who showed closeness, truthfulness, and responsiveness, while intrapersonal knowledge was attributed through coaches' adaptability. Inmates identified all four possible outcomes and emphasized that sensitivity, authenticity, energetic demeanor, physical appearance, and athletic skills are crucial qualities for an effective coach in a prison.

**Discussion:**

The balance between an authentic and energetic demeanor, including a certain degree of strictness, emerges as a key factor in effective coaching within the prison context.

## 1 Introduction

Sports coaches can facilitate or hinder athletes' progress (1), both in athletic performance (2) and personal development (3). Personal development is particularly desirable when coaches deal with socially disadvantaged population groups (4). Due to social isolation and the manifold negative consequences associated with incarceration (5), inmates can be described as such a population group.

Sports have been found to play a significant role in the development and daily life of inmates. Participating in sports activities can lead to improvements in both physical health, such as increased fitness and reduced health risks (6), and mental health, including reduced symptoms of depression and anxiety (7). Sports also provide a constructive outlet for managing aggression and anger, allowing inmates to positively channel these emotions (8). Engaging in sports helps inmates to build self-confidence by achieving personal goals and overcoming challenges (9). Additionally, involvement in sports promotes the acceptance of societal values and adherence to rules, fostering a sense of discipline and respect (10)—aspects that are especially important as part of inmates' rehabilitation process. Furthermore, sports activities facilitate the formation of social relationships, offering inmates opportunities to interact and collaborate with others, what is crucial for their social development (11). In summary, sports in prison provide to a wide range of health and social benefits and support inmates in their rehabilitation process.

Sporting activities require qualified and effective coaches (12). It is unclear whether inmates are able to assess the effectiveness of coaches in the same way as athletes outside of the prison, and whether they perceive the same aspects as relevant. Joint exercises with other inmates are considered more promising for inmates' rehabilitation than individual practice (13). Additionally, guided sports programs by coaches are characterized by higher regularity and higher effectiveness (14).

Besides two studies from Spain and Germany, there is no further research on coaches in prisons (and their effectiveness). In the Spanish study (15), social education students who provided sports programs in a prison shared their experiences in a diary. However, the study focuses on student learning rather than coaching. The German study (16) examined the coaches' experiences in providing sports programs in prison, suggesting that specific education is necessary to adequately prepare coaches for the prison context. Problematically, the study suggests that previous coaching experiences in other contexts offer limited preparation for the distinct obstacles encountered within prisons. In the prison context, coaches face challenges such as the inmates' tendency to reject weaknesses, poor self-assessment, and over-confidence. Social factors (e.g., disputes, theft of equipment) but also external factors (e.g., other inmates observing the sporting session) further complicate the coaching process (16).

While these studies were focused on the providers' perspective (e.g., coaches), this study investigates inmates' perspective on sport coaches. In line with previous research in sport coaching, the study is based on Côté and Gilbert's (17) model of effective coaching. Effective coaches need knowledge (i.e., professional, interpersonal, and intrapersonal) and should help athletes achieve outcomes (i.e., sport-specific competence, confidence, and connection to others). Furthermore, effective coaches need to consider the specific coaching context (17). Examining inmates' perspectives on sports coaches is crucial for enhancing the effectiveness of rehabilitation programs. Inmates face unique challenges such as social isolation and an increased risk of mental health issues. Targeted sports programs can address these challenges, underscoring the importance of focused research in this area (18). Conditions for the success of such sports programs reflect, for example, adequate didactic preparation and the implementation of reflection discussions (19).

## 2 Theoretical framework and literature review

**Figure 1:**
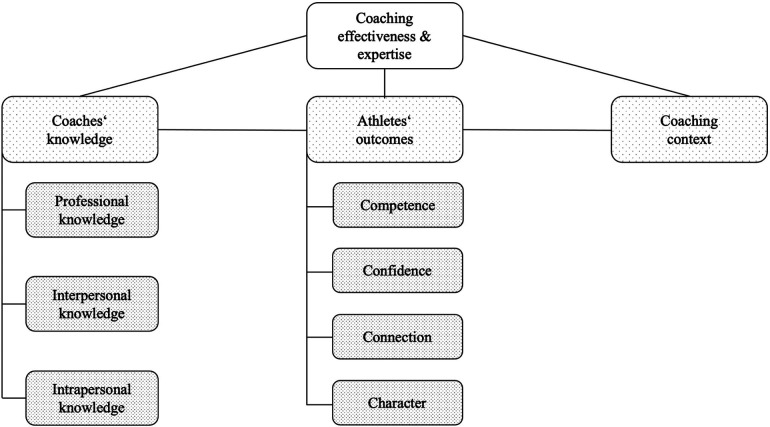
Coaching effectiveness and expertise (own illustration according to (17).

According to Côté and Gilbert's (17) integrative definition, effective coaching involves “the consistent application of integrated professional, interpersonal, and intrapersonal knowledge to improve athletes' competence, confidence, connection, and character in specific coaching contexts” [(17), p. 316]. While this framework provides a comprehensive overview of coaching effectiveness, its applicability in special contexts such as prisons warrants further scrutiny. The framework refers to three central factors of coaching effectiveness: Coaches' knowledge, athletes’ outcomes, and the coaching context (17). The framework has already been used in previous studies, for example in soccer (20) and youth development through sport (21). However, the distinct challenges in the prison setting, such as security concerns and rehabilitative goals, necessitate a critical evaluation of how this framework can be applied. Figure 1 displays all factors of the framework, and they are explained in more detail in the following sections.

### 2.1 Coaches' knowledge

Behaviors, dispositions, education, and experiences of a coach are important determinants of success and can be summarized as coaches' knowledge. Effective coaches need professional, interpersonal, and intrapersonal knowledge (17). Professional knowledge includes declarative knowledge in sports sciences, sport-specific, and pedagogical knowledge with accompanying procedural knowledge (22). All of these dimensions were already examined in competitive sports with formalized training for coaches (23), but little attention has been paid to the professional knowledge of coaches in prison. Devís-Devís et al. (24) identified a limited degree of professionalization in prison's sport provision, suggesting that coaches in these settings may not receive the same structured training as coaches in other settings and contexts. This finding suggests a significant gap in training programs, questioning whether current educational frameworks adequately prepare coaches for the unique demands within the prison environment. In order to provide a successful sports program, Wicker et al. (16) found that coaches in prisons need specific knowledge and that they should consider special success factors (e.g., involvement of inmates, provision of a sport program by at least two coaches with strong personalities).

Interpersonal knowledge reflects coaches’ ability to interact regularly with their athletes and use appropriate and effective communication strategies. Given that the coach-athlete relationship is described as the heart of coaching (25), coaches need to consider athletes' age, skill level, and social context (17). A review by Langan et al. (26) indicated that improving coaches' interpersonal effectiveness can enhance athletes' behavior, affect, and cognition. However, research indicates that the effectiveness of interpersonal skills can vary depending on the context and the individual athlete's needs (27). In prison, the development of interpersonal relationships is characterized by a difficult balance between closeness and distance to inmates or between trust and authority (16). This balance poses a challenge to traditional coaching methods and calls for innovative strategies to maintain effective communication without compromising safety or authority. Even outside of sports, effective interpersonal communication is seen as a major challenge in prison (28). Cushion (29) suggests that interpersonal knowledge might take precedence over professional knowledge in certain contexts, such as non-competitive or recreational settings. Accordingly, it is important to investigate whether this suggestion is also valid in a prison context. In prisons, trust and relationship-building are crucial for effective rehabilitation (30). Therefore, interpersonal knowledge may significantly enhance coaches' ability to engage with inmates, build a relationship with them, and address their unique needs.

Intrapersonal knowledge refers to coaches’ ability to introspect, reflect, and revise practices (17). Intrapersonal knowledge is difficult to measure because it is not consciously expressed in the coaches’ daily practice (31). While often considered crucial, reflective practices in rule-based, highly structured environments may be framed differently due to external constraints. Some studies indicate that intrapersonal knowledge may be less critical in such highly structured settings (32). In highly structured, rule-based environments, the focus is often on consistency, efficiency, and adherence to external standards (33). These settings prioritize predictable outcomes and compliance over personal insight, reducing the perceived need for individual reflection. Accordingly, it might be possible that coaches' intrapersonal skills are less important from the inmates’ perspective. In a qualitative study about coaches' experiences in a German prison, the coaches reflected on their personality and behavior after the training sessions, while trying to demonstrate confidence during the sessions (16).

### 2.2 Athletes' outcomes

An effective coach should contribute to the athletes' outcomes. Côté and Gilbert (17) conceptualized the outcomes with the four C's: competence, confidence, connection, and character. Competence represents different performance indicators, such as sport-specific technical and tactical skills, improved health, or healthy training habits (34). Inmates' participation in sports had a positive effect on their fitness level [e.g., (6)]. Confidence is characterized by an internal sense of positive self-worth, with the coach-athlete relationship as important determinant (35). Sports programs in prisons helped to decrease inmates’ perceived feelings of stress and increased their subjective well-being and level of self-confidence [e.g., (36)]. Social relationships with other people are referred to as connections. Previous studies underlined the relevance of sports in prisons for improving social relationships and communication between inmates [e.g., (37)]. Character development involves cultivating qualities such as integrity, empathy, and responsibility. Effective coaches play a crucial role in this process by modeling these traits and fostering an environment that encourages personal growth. In the context of prison sports, coaches can significantly influence inmates by promoting non-criminal behavior, discipline, and tolerance [e.g., (7)]. Additionally, positive relationships with coaches can provide inmates with valuable role models and support systems (15), further enhancing their character development.

### 2.3 Coaching context

Coaching contexts are the unique settings in which coaches try to improve athlete outcomes, and effective coaches must be aware of the overriding sports context in which they work (38, 39). However, there is evidence that coaches lack knowledge about the prison context because it is neither part of sports science majors nor coaching licenses (16). In addition to protecting the public from further crimes, prisons serve the purpose of rehabilitation (40). These objectives are related to two key challenges for coaches in prisons (22): Coaches must ensure the safety of themselves and inmates because prisons are “violent places” [(41), p. 1159], and coaches must adapt their strategies to align with the broader rehabilitative goals (42). These challenges underscore the necessity for a deeper understanding of how coaching methods can be adapted to meet the specific demands of the prison environment effectively. For instance, coaches must develop strategies that prioritize safety by implementing structured and controlled training environments to mitigate the inherent risks of violence and ensure a safe sport (43). Additionally, they need to incorporate rehabilitative elements into their coaching, such as emphasizing teamwork and personal growth, to align with the broader rehabilitative goals of the institution (40).

### 2.4 Research questions

According to the theoretical framework and the state of research, the study addresses three research questions: (1) How do inmates perceive the knowledge of their coaches? (2) What outcomes do inmates attribute to the actions of the coaches? (3) Which qualities do inmates perceive as important for coaches in the prison context?

Our study addresses existing research gaps by specifically examining the applicability of Côté and Gilbert's (17) coaching framework within the unique context of prisons. By investigating inmates' perceptions of their coaches' knowledge, the outcomes they attribute to coaching, and the qualities they deem important for effective coaching in prisons, our study aims to provide insights into how coaching methods can be adapted for this specific setting. This study not only contributes to the theoretical understanding of effective coaching in non-traditional contexts but also offers practical implications for developing coaching programs that support inmate rehabilitation and safety.

## 3 Methods

To ensure a comprehensive understanding of the research process, it is important to address epistemology, positionality, and reflexivity. The epistemological stance of this study aligns with a constructivist approach, recognizing that knowledge is co-constructed by researchers and participants (44). This approach is particularly relevant in the prison setting because it acknowledges the complex and subjective realities of inmates' experiences. By adopting a constructivist approach, the study aimed to value the inmates' voices and perspectives, recognizing that their insights on sports coaching are shaped by the social and institutional context. The researchers encouraged open dialogue during the interviews, which allowed inmates to share their experiences and insights without being judged. This approach was chosen because it facilitates a deeper understanding of the nuanced and personal experiences within the complex prison system, thereby enriching the study's findings with authentic, co-created knowledge.

Positionality was acknowledged by the researchers, who actively considered how their own backgrounds (e.g., education, socio-cultural contexts) might influence data interpretation (45). The research team comprised individuals with backgrounds in pedagogy, exercise physiology, sports science, and sociology, each bringing unique perspectives to the study. For instance, those with a background in exercise physiology reflected on their understanding of structured training environments, while those with a sports science and sociology background considered broader social dynamics within the prison setting. Researchers also reflected on how their own (external) perceptions of authority and rehabilitation might differ from those of the inmates, which helped in maintaining an open approach during the data analysis procedure. By recognizing these diverse backgrounds, the team was able to reflect potential biases in interpretations.

Reflexivity was actively ensured throughout the study, with researchers continuously reflecting on their interactions with the participants and the data. This approach included that the interviewers took notes after each interview to capture initial impressions and emotional responses. Based on these notes but also during the data analysis process, researchers engaged in regular team discussions to critically evaluate their assumptions and interpretations. By remaining aware of their influence through these practices, researchers ensured that interpretations remained grounded in the inmates' perspectives and experiences. This reflective approach has not only minimized bias but also enhanced the credibility and authenticity of the findings (46).

### 3.1 Research context

The study was carried out as part of a larger project and involved collaboration between a specific branch of a German prison and a university. This specific branch has capacity for 60 male inmates and sports activities in the branch were restricted to self-guided strength training or casual soccer matches before the collaboration. The branch is characterized by an open system, allowing inmates to leave during the day for school or work. Afterwards, inmates must return to the prison. Open prisons typically house inmates who have demonstrated good behavior and a lower risk of escape or re-offending.

The sports program provided by the university was conducted from November 2020 to June 2021 and comprised four programs, each lasting for 6 weeks. In each program, participants engaged in three training sessions per week, resulting in a total of 18 training sessions for each program. The first program concentrated on enhancing endurance (47), the second program on strength. The third program included dance-based martial arts training (48), while the last program involved a soccer training program (49). Participation in the programs was voluntary.

In total, twelve external coaches were responsible for delivering the programs. All these coaches were either currently enrolled in or had previously completed a higher education program related to sports. Collectively, they hold multiple coaching qualifications and have coaching experiences in multiple sports contexts (e.g., school, community sports club, gym, university sports). None of the coaches has worked with inmates before these programs; they were not specifically prepared in advance of the programs nor were they aware of the theoretical model.

The participation of numerous coaches in the program, despite having only 43 inmates, was designed to provide a diverse range of expertise and coaching styles, enriching the overall learning experience for the inmates. Each coach brought unique skills and perspectives, which aimed to address different aspects of athletic training and rehabilitation.

### 3.2 Data collection

In total, 43 inmates participated in the four programs. Three inmates participated in all four programs, and two inmates in three out of four programs. Only these five inmates were asked to participate in an interview about the perceived effectiveness of the coaches. This criterion-guided case selection represents an extreme group sampling approach that ensures comprehensive insights from the interviewees (50). Table 1 displays socio-demographic information on the interviewees. They were between 23 and 42 years old, the duration of detention ranged between 19 and 83 months, and they had different sporting backgrounds.

**Table 1:** Interview participants: socio-demographic information.

Interviewee	Sport program participation	Age (years)	Duration of detention (months)	Sport participation before detention
1	1, 2, 3, 4	26	19	Soccer (sports club); fitness & martial arts (gym)
2	1, 3, 4	23	35	Soccer (sports club); fitness (gym)
3	1, 2, 3, 4	25	19	Soccer (sports club); fitness (gym)
4	1, 2, 3, 4	25	23	Running; fitness (gym)
5	1, 2, 3	42	83	Soccer (sports club)

In November 2021, the five inmates were interviewed using a qualitative semi-structured approach. The structure of the interview guide resulted from the theoretical model of effective coaching (17) and included questions related to coaches' expertise and competence, inmates' perception of their own development, and the relevance of context-specific coaching skills. The interview guide included a variety of questions aimed at capturing inmates' perceptions of each knowledge and outcome area, rather than directly addressing theoretical assumptions. This approach facilitated the gathering of nuanced insights into how inmates experience and understand different knowledge and outcome domains. By following best practices in qualitative research, which suggest that a diverse set of questions can elicit richer, more comprehensive data (51), the interviews were structured to be conversational and adaptive. This approach encouraged inmates to share their experiences, resulting in deeper insight into their perspectives.

The interviews were led by the second author and an undergraduate student, since the lead author was one of the coaches. Both interviewers were unknown to the inmates before the interviews to allow anonymity and avoid social desirability bias (52). The interviews were conducted in German language and in person in a visitors’ room in the branch. The interviews were recorded, transcribed verbatim, and translated into English by the lead author, the second author, and another person not involved in the study. Before the interviews, all five inmates indicated their consent to participate, and an institutional ethical approval was granted by the hosting university under the reference number EUB-2022-149.

### 3.3 Data analysis

The interviews were analyzed by the first and second authors using directed content analysis (53) and MAXQDA software. During the process, repeating aspects that are relevant to the research questions were systematically extracted from the material and summarized in categories (53). This procedure allows the combination of a theory-based (deductive) and inductive approach that supports and extends an existing theory about the phenomenon under analysis (54). Thus, existing theoretical concepts and findings from previous research are defined in advance to guide the analysis, while new insights and categories can also be drawn from the material. The aim was to gain crucial meaning from complex interview data (55). Interview data from inmates present unique complexities due to factors such as diverse cultural backgrounds and varying levels of education, which can create, merged with forms of mistrust, communication barriers (56). These elements, combined with the psychological and emotional nuances inherent in their narratives, require a nuanced analytical approach (57). Qualitative research can address these challenges by providing flexible methodologies that can capture and interpret the rich, layered meanings within data. This approach is critical for generating insights that are both comprehensive and context-sensitive (58).

The directed content analysis followed the procedure described by Mayring (53). The deductive categories were based on the theoretical framework of Côté and Gilbert (17), highlighting eight main categories: (1) professional knowledge, (2) interpersonal knowledge, (3) intrapersonal knowledge, (4) competence, (5) confidence, (6) connection, (7) character, and (8) context.

In the first coding round, the authors familiarized themselves with the material, assigned text fragments to the categories, and marked possible anchor quotes independently from each other. After this initial round, the authors compared and discussed their assignments to the categories, resulting in improved coding rules since the distinction between some categories was not sufficiently clear. For the coaching context, the authors discussed the presence of contextual references in all categories. This is particularly the case in the two categories interpersonal knowledge and connection. To avoid double coding, only qualities that inmates perceive as especially important for coaches in the prison context were coded as context.

Both authors did a second round of analysis individually and compared the assignment of codes afterward. In the next step, inductively developed subcategories were added to the category system as further aspects seemed relevant to understanding coaching effectiveness in prison. For the coaching context, the subcategories' sensitivity, demeanor, appearance, and abilities were inductively developed.

## 4 Results

**Figure 2:**
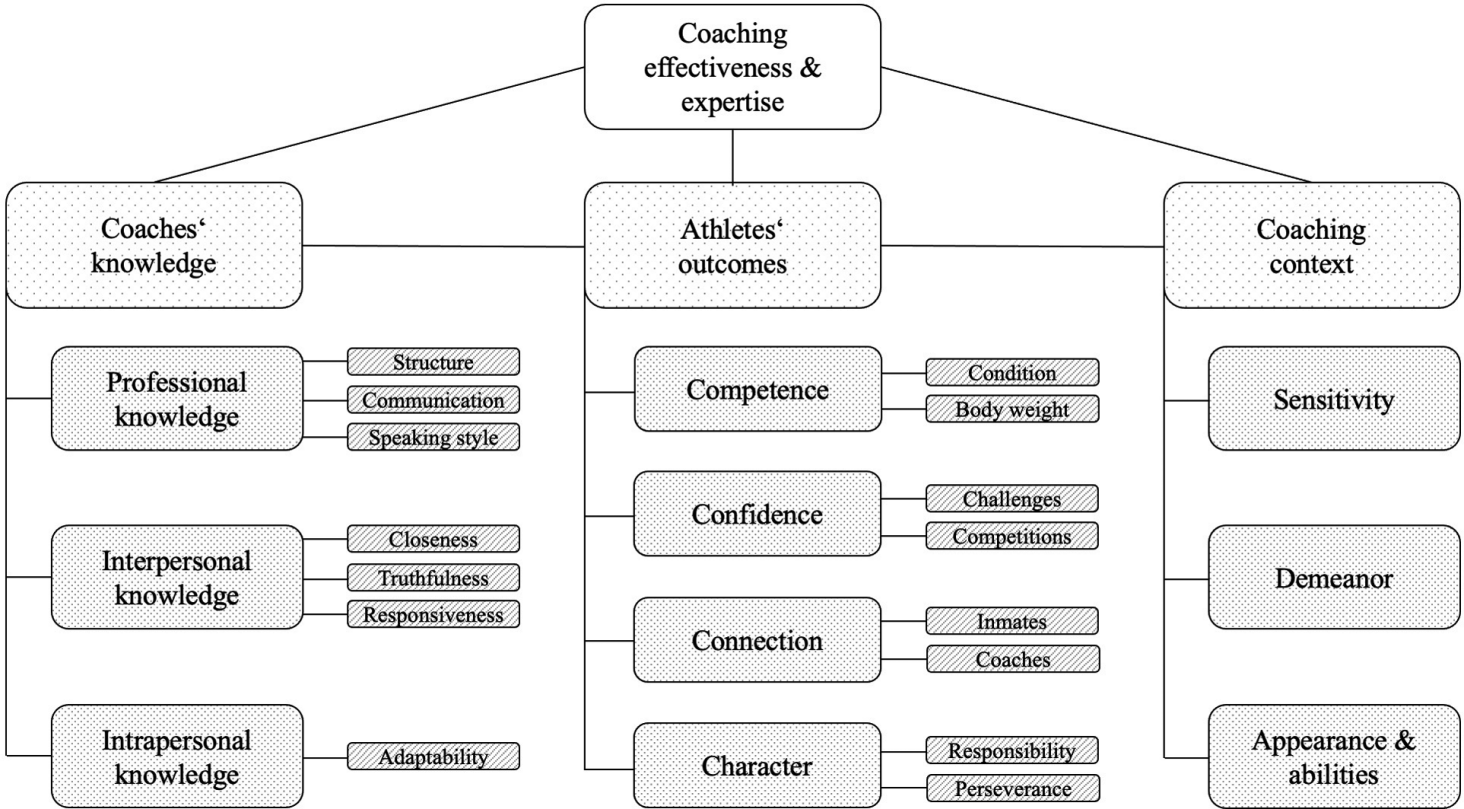
Category system for coaching effectiveness in the prison context (own illustration).

Figure 2 shows the final category system for the analysis. The results are presented by categories and subcategories.

### 4.1 Knowledge

#### 4.1.1 Professional knowledge

Inmates perceived professional knowledge in their coaches through structured training and clear, transparent communication. The following quote supports this notion:

“The endurance training was always very structured. [.] He [the coach] also explained it. And we understood it. And he also explained why that was the case. And I felt that he knew a lot about it” (B1, Pos. 48).

Likewise, another inmate referred to the coaches' communication style:

“Because they knew exactly what they were doing. Sometimes they explained what effects it [the training] has. On body, perhaps also on the psyche. I thought that was very cool” (B3, Pos. 60).

The inmates determined professional knowledge not only by the content of the communication but also by the way how coaches spoke (B5, Pos. 44). The way of speaking was associated with educational attainment or intelligence (B2, Pos. 57).

“You always noticed it when they were talking. Good friends of mine are also high school graduates. So, ‘talking intelligently’ sounds stupid. But I just know it” (B3, Pos. 64).

One interviewee recognized a high level of athletic talent and physical abilities among the coaches as an indicator for coaches' professional knowledge.

“Appearance […] usually shows me whether someone is talented in sports, or whether he can do something or not […]. For example, in weight training, [the coaches were] a bit more stable in terms of physique. With the guys from endurance training, they weren't as stable, but you realized, OK, they can run. They can run and talk without breathing twice in a row. Yes, and that showed me that it is about more than just performance. Acting as if they were some kind of coaches” (B2, Pos. 51).

In summary, the inmates perceived that all coaches had professional knowledge. The main coaches of the endurance and dance programs were especially highlighted as “real professionals” (B4, Pos. 30), because the inmates perceived that these coaches have “long time experience” (B4, Pos. 30).

#### 4.1.2 Interpersonal knowledge

Interpersonal knowledge was characterized by the coaches' ability to interact with inmates in a respectful and non-judgmental manner. This approach fostered feelings of comfort and acceptance, as expressed by one inmate:

“They did not look at us as like we were scum or something just because we made a mistake. That they think we must keep our distance. No, they did the opposite. […] Well, they also criticized us. They always gave us their opinion. Some did not think that was great. But in my opinion, it is exactly the right thing to do” (B2, Pos. 18).

This opinion was shared by all interviewed inmates. All of them appreciated that the coaches tried to establish regular interaction (without distance) and communicated truthfully. In this climate of closeness and truthfulness, the inmates felt comfortable:

 “They were responsive for me. They were responsive for everyone. […] You felt like you were in good hands” (B5, Pos. 32).

The inmates did not observe the described feeling of regular interaction for all coaches. One inmate attributed this fearful behavior to an overly cautious approach resulting from a tense mood or anxiety among the coaches in the second program:

“During strength training, I noticed that the boys [the three coaches] were a bit more anxious and cautious. They did not really notice us and were more afraid. And they did not need to be. After all, we are only in a prison. OK, I understand that, but we are people, too” (B2, Pos. 22).

According to the inmates, most of the coaches communicated appropriately. The communication was “factual”, “calm”, and “without discussion” (B3, Pos. 133).

#### 4.1.3 Intrapersonal knowledge

Intrapersonal knowledge was demonstrated through the coaches' self-awareness and adaptability in response to the dynamic prison environment. The inmates described unplanned organizational changes within the training during one session when the coaches noticed the long waiting time and lack of activity of many inmates. This adaptability was illustrated when a coach adjusted training sessions based on real-time observations:

“In strength training, for example, we had four exercises. They [the coaches] changed that [the number of stations] because they realized that it was not working. Twelve or 13 people had to wait. That took a bit of time. After that, they changed it a bit” (B4, Pos. 82).

Intrapersonal knowledge was also recognized in another situation, in which one of the coaches shared his reflection about a problematic situation with the participants. The situation was described by an inmate:

“There was a situation in the endurance intervention when I1T1 got loud once. Everything went wrong. And he realized that the guys were no longer there [with their heads]. […] And then he told me afterwards in a conversation […]: “You will not believe it, I got loud. […] But I was so disappointed, and things were going so well” (B5, Pos. 42).

### 4.2 Athletes' outcomes

#### 4.2.1 Competence

The programs led to noticeable improvements in inmates' physical and motor skills, indicating enhanced competence. Inmates participated in assessments pre- and post-intervention, as one inmate described:

“We weighted ourselves. We did exercises that we also did before the intervention, before we started. And then they [the coaches] said that we would do the same after the intervention just to see if you have developed or not. Yes, and in the end, many have developed, physically but also the endurance” (B2, Pos. 93).

Many inmates improved their physical condition as well as their motor skills during the programs. The physical development was mainly determined by a reduced body weight (B2, Pos. 65; B3, Pos. 110; B5, Pos. 62). Another inmate differentiated development based on the specific requirements of the programs as well as his level of entrance:

 “In the third intervention, capoeira. You did not notice the difference in your body. You noticed; I think it was concentration. That is why I could not notice it, because I was, and I am good. With the first intervention I did not know that I have good endurance. […] That means I was the last one to give up in the test. […] That was when I noticed that my endurance improved, not so much in the second intervention because I did strength training beforehand. […] I never played soccer, except on PlayStation. That is where I started. The first thing the coaches noticed was that it was bad. […] And I noticed that I improved in the fourth intervention in soccer” (B2, Pos. 93).

The development of motor skills was evident across the four programs. Due to his positive development of fitness or endurance, one inmate has continued running and developed new habits after the program ended:

“My endurance, for sure. I still run a few laps, even though I do not do any sport at the moment. I always run in between” (B3, Pos. 100).

#### 4.2.2 Confidence

The sporting programs contributed to building confidence among inmates, allowing them to overcome personal challenges and fears. One inmate reflected on this newfound self-assurance:

“Well, I tore my cruciate ligament a year and a half ago. I did not do any sport for a year and a half, nothing at all. And I was also scared because I knew that if I twisted my ankle, it would not end so well. That is why I talked to I1T1 several times. […] That also gave me a bit of self-confidence. Confidence in myself that I am going to make it” (B2, Pos. 34).

Through conversations with the coach, the inmate received guidance and reassurance, which helped to alleviate his fears and build self-confidence. This interaction highlights the coach's role not only as a physical instructor but also as a mentor and supporter, providing the encouragement needed for the inmate to believe in his abilities. The coach's engagement and personalized support were instrumental in fostering the inmate's self-assurance, demonstrating the importance of a supportive coach-athlete relationship in the process of confidence building.

Other inmates also reported positive emotions related to the sporting programs. However, these can rather be described as moments of joy or anticipation, which did not influence self-esteem. Though these emotions do not directly increase self-esteem, they create a supportive environment for confidence-building, demonstrating that emotional well-being is a component of overall confidence.

“You were just distracted. You looked forward to it. If you had a bad day, you could say, yes, the soccer group is coming back today” (B1, Pos. 100).

Competition is a key factor in developing confidence, as it encourages participants to push their limits and recognize their capabilities, thus fitting well within the theme of confidence-building. The opportunity of comparison or competition with others was highlighted as motivational by another inmate:

“There was competition. Who wins, who loses? That is fun too. It is motivating. It motivated me personally. I mean, I always want to win. […] Everybody went full throttle to make sure they could win” (B4, Pos. 12).

One inmate reported that participation in the programs changed his attitude toward sports and developed an inner satisfaction in doing sports on his own. This change in mindset and the resulting satisfaction contributes to a deeper sense of confidence, as the inmate finds value and accomplishment in actions, aligning with the broader theme of confidence development.

“But I notice already […], you have done something good for yourself. […] I realize that I am satisfied for myself when I have done something. Well, I did not enjoy sports at the beginning because it was just so difficult. Now I think, I am off work and then I go there [do sports] afterwards” (B5, Pos. 70).

#### 4.2.3 Connection

The programs fostered connections on multiple levels, both among inmates and between inmates and coaches. These relationships developed over time, as inmates began to see the coaches as allies rather than authority figures:

“But I have to say, the first times when we came together like this. The first unit of the intervention with I1T2 and I1T1, endurance. And they also came across as really nice. We also had some kind of a bond with them. And we were also the sports group here. That was also really fun” (B5, Pos. 28).

Social relationships were evident on different levels. First, between the coaches and the training group, and second, between the inmates within the training group. On both levels, the inmates experienced making new bonds. However, the relationship with the coaches developed over time:

“In the beginning, we called each other by our last names. And then at some point, they said: ‘Yes, guys, you can call us by our first names’. We just gave them the feeling that we are in prison, but that they do not have to have the feeling that we are criminals or that we do not follow the rules here” (B2, Pos. 16).

Building trust obviously followed a bidirectional process. Both sides displayed normal and respectful behavior, which gradually led to more communication and openness.

“[…] They also told us about their lives, asked us questions, and gave us honest answers. […] And I think there was also a bit of gratitude, and they also opened. […] I think that is always important when you are treated as an inmate […]. Anyone who still sees you as a human being rather than just as: ‘Oh, what have you done?’” (B5, Pos. 28).

The most important thing for the inmates was that they were seen as human beings by the coaches. In contrast, a higher intention was needed for building connections between inmates. Thus, the soccer program was deliberately designed to promote social integration and cohesion. This intention had been recognized by the inmates.

“We also had some exercises on teamwork and communication. And I think that's how you noticed that they had a handle on how we acted as a team. And not to separate someone who then stands there alone” (B1, Pos. 60).

There were also situations in the other sports in which the inmates had to support each other:

“We always did teamwork in between […]. I didn't know one person at all. I did some exercises, I got to know him, and we had fun throughout the exercise. That helped a little bit. Just getting to know the athletes, the inmates with each other. Living together” (B4, Pos. 64).

The closer relationship during the programs resulted in extended communication in the everyday life of inmates in prison.

“We were just a solid group that pulled through from start to finish. And of course, when you saw them, you started to talk to them more. For example, you asked them: ‘Are you coming to sports or not?’” (B2, Pos. 38).

However, the inmates clearly put the development of relationships into perspective. They moved closer together, but nothing had changed in terms of friendship (B1, Pos. 110 & 112). Trust had developed, but not so much “that one could let oneself go” (B2, Pos. 32). Even after the sport programs, friendships in prison were not considered as desirable.

“But friendship? Friendships are very dangerous anyway. Anyone who thinks they must make friends, I think, is also very out of place” (B5, Pos. 82).

#### 4.2.4 Character

Participation in sports helped inmates to reconnect with values such as respect and integrity, contributing to character development. One inmate found this process transformative:

“As I said, I have been pretty criminal for years, but I always tried to be reasonable, decent, and respectful. […] In these interventions, I have been able to brush up on that a bit. I found myself again” (B3, Pos. 163).

This inmate was able to get back to values of respect and integrity through sports. Such social values in sports were not only met in self-perception but also related to other inmates. The inmates experienced feelings of responsibility and empathy for each other. For example, this inmate reported how he supported someone during the training session.

“I grabbed one or two who were not so blatantly athletically gifted, who could not get through because of their body weight. Then I simply exercised with them. I said: ‘We also exercise together to maintain the bond’. And to motivate them further” (B2, Pos. 38).

Responsibility was not only taken for other inmates but also for oneself. The development of personal responsibility is expressed, for example, in not giving up and finishing.

“The interventions have taught me not to give up. I am a person whom when I start something, I want to finish it. […] In the end, I am proud that I pulled it off” (B2, Pos. 113).

### 4.3 Coaching context

Based on their previous or current experiences in sports, the inmates described three special qualities for coaches in the prison context: Sensitivity, authentic and energetic demeanor, and physical appearance and sporting abilities.

“You might have to be a bit careful with your words, sentences, or announcements since people react differently. Otherwise, it is quite normal. I think it is more a matter of sensitivity. How to assess people, how far you can go” (B1, Pos. 148).

Being sensitive as a coach and constantly evaluating your athletes' moods can be seen as a regular requirement for a coach. At the same time, it was clear that not only what was said had an impact, but also how it was communicated.

“Be relaxed, not tense. Because we realize, OK, they are scared. And then a lot of people probably take advantage of that. It is like: ‘Okay, they are scared, then they will not listen to us. So, we can just do whatever we want’. No. It is important to be proactive. […] Always follow through and be how you are. That is the most important thing here” (B2, Pos. 155).

According to the inmates, such an authentic and energetic demeanor includes acting actively and strictly.

“Do not show weakness […]. Normal, respectful, of course. But also, a bit stricter. That is quite important” (B3, Pos. 204).

However, the inmates did not primarily base their respect and recognition for the coach on interpersonal competencies. Instead, they highlighted the importance of the coaches' physical appearance and sporting abilities:

“The visual is important. With I1T1, for example, he is quite small and they [the other inmates] just perceived that, but when he started running, […] he was faster than all of them, and that is what they needed. Someone who is the leader, who can do it, but also looks like he can do it. That is important. It is like being in the jungle, eat and be eaten. A shark is not afraid of a goldfish” (B5, Pos. 78).

## 5 Discussion

The study aimed to investigate inmates’ perception of effective coaches in prisons, drawing on the framework of coaching effectiveness by Côté and Gilbert (17). While this framework provides a robust foundation for understanding coaching effectiveness, its application in the unique context of prisons may contribute to the identification of specific challenges and dynamics inherent in working with incarcerated populations. The results are discussed in the order of the three research questions.

The inmates perceived the coaches obtained all three types of knowledge by identifying specific characteristics in the coaches' behavior. The inmates recognized professional knowledge in coaches who demonstrated clear training structures and communication of transparency and understanding. This finding is in line with Shute (59), who reported that clear structures and communicated expectations have a positive impact on the learning success of students. Accordingly, learners benefit when they understand the course of the lesson and know which goals must be achieved. The importance of transparent communication is undisputed, both in educational science (60) and in coach research (61). Deci et al. (60) emphasized that transparent communication regarding goals and purposes promotes the intrinsic motivation of learners. According to Szedlak et al. (61), coaches can influence athletes' development through effective instructions and communication. Interestingly, inmates primarily mentioned communication as an indicator of professional knowledge, although it is rather part of the interpersonal knowledge in the framework of Côté and Gilbert (17). This overlap may suggest that inmates equate clear and effective communication with expertise, potentially due to the unique power dynamics and trust issues present in a prison setting. Further research could explore how these perceptions influence the overall effectiveness of coaching. Furthermore, the overlap can be explained by the fact that, in addition to the content of the communication, the language style, i.e., the manner of expression, was the main criterion assessed. The language register is described—also by the inmates—as educational or academic. According to Quasthoff (62), academic language is linked to the knowledge and competence of the speaker.

Interpersonal knowledge was valued, with inmates expressing a preference for coaches who allowed emotional closeness, communicated honestly and calmly, and fostered a comfortable and supportive interpersonal climate. In line with the coaches' assessments investigated by Wicker et al. (16), the development of interpersonal relationships in prisons is characterized by a balance between closeness and distance. The delicate balance between closeness and distance poses both opportunities and risks. While fostering trust and openness can enhance learning, it may also blur boundaries that are critical in a correctional environment. This balance warrants careful navigation to avoid dependency or boundary issues. Otherwise, the social qualities for the coaches correspond to those in other coaching contexts outside of the prison. For example, these results are in line with Carson et al. (63), who highlighted that strength and conditioning coaches should generally create a supportive environment.

Intrapersonal knowledge was recognized since coaches demonstrated adaptability by making real-time adjustments to training sessions. That is in line with Collins and Collins (64), who found that the requirements of being adaptive and flexible can be met through a careful process of professional judgement and decision-making based on context-appropriate knowledge (64). Although adaptability is a valued trait, the prison environment presents unique challenges that may limit a coach's ability to implement changes effectively. Factors such as institutional policies and the unpredictable nature of inmate behavior must be considered.

The predominantly positive perception of the coaches' knowledge by the inmates might be explained by the coaches' external status and their (predominantly) high level of qualification and experience. Inmates may perceive and evaluate coaches employed as sports officers in the prison differently than external coaches.

Turning to the outcomes stimulated by the coaches, inmates perceived effects related to all four dimensions (i.e., competence, confidence, connection, and character). Inmates reported improved competence, including enhanced physical condition and reduced body weight, supporting the effectiveness of sport programs in prison in quantitative terms [e.g., (6)].

Confidence was also improved since inmates perceived that they were able to overcome physical challenges. Furthermore, increased levels of motivation derived from the implementation of performance tests and opportunities for competition. Once again, this study indicates that sports offer inmates the opportunity to perceive themselves as capable of achieving specific outcomes, which, in turn, can contribute to forming the inmates' identity (65). In line with Lleixà and Ríos (15) and Dransmann et al. (37), coaches play a fundamental role in fostering connections both between coaches and inmates and within the inmates’ group. However, coaches should primarily focus on the coach-athlete relationship rather than on improving connections between inmates, since a prison was not considered the right place for real friendships. Moreover, positive changes in character were perceived by the inmates, for example through rediscovery of values (e.g., respect, integrity). Feelings of responsibility and empathy for fellow inmates were developed, and inmates emphasized the importance of personal responsibility and perseverance as key lessons. Individual development regarding perseverance, respect, and teamwork cannot only be achieved in competitive youth sport (66) but also in a prison context. While positive outcomes were reported, their long-term sustainability post-program remains uncertain. Factors such as continued support, follow-up interventions, and the broader prison environment play crucial roles in maintaining these benefits (67).

Within the prison context, inmates perceived specific qualities as significant for coaches. Inmates stressed the importance of coaches possessing sensitivity in their interactions and carefully choosing words to navigate diverse reactions. Szedlak et al. (61) also highlighted that a coach should be sensitive, but more in the sense of being concerned and mostly focused on the personal needs of their athletes. Accordingly, sensitivity and a careful approach are a particular challenge within the prison setting. Inmates come from different backgrounds and may have experienced post-traumatic stress disorder and violence (68). Therefore, sensitivity is especially important for coaches in prisons. This finding underlines the importance of effective coaches who are competent, empathetic, and aware of the diverse backgrounds and experiences of inmates.

An authentic and energetic demeanor, including being strict, when necessary, was deemed crucial for an effective coach in prison. There is evidence that the authenticity of a coach and the congruence in the coach-athlete relationship influence rates of burnout in collegiate athletes (69). At the same time, the inmates consider a certain degree of strictness and authority as necessary for sports coaches in prison. Such values are related to a paternalistic and autocratic leadership style of coaches (70). According to Jin et al. (71), democratic leadership behaviors had a more positive influence on coach-athlete relationships, and athletes’ motivation and satisfaction in Chinese collegiate athletics than autocratic leadership behaviors. Therefore, a (difficult) balance of energy and strictness can promote effective coaching in prison by combining positive motivation with clear expectations.

Inmates primarily based their respect on coaches' physical appearance and the demonstrated sporting abilities, highlighting the importance of demonstrating the physical skills needed for a sport. The reliance on physical appearance as a marker of respect raises questions about the depth of inmates' understanding of competence. This perception may perpetuate superficial evaluations of ability and detract from recognizing other critical competencies. On the other hand, this finding is in line with a study from the fitness sector, suggesting that customers perceive more muscular personal fitness coaches as more knowledgeable and competent than their nonmuscular peers (72). In addition, elite-level athletes reported that they appreciate coaches who were former athletes (73).

In summary, the findings of this study have both theoretical and practical implications for sports programs in prison. Comparative analyses with other educational or rehabilitative environments (such as closed prisons or female inmates) would provide valuable insights into the transferability of perceived coaching effectiveness. Additionally, longitudinal studies would help to explore specific coach characteristics, particularly leadership styles.

In conducting interviews with inmates, we encountered several communication complexities due to diverse cultural backgrounds and varying levels of education. To address these challenges, we simplified language to ensure clarity, maintained confidentiality to build trust, and clearly communicated the study's purpose. Active listening techniques were employed, and participants were encouraged to ask questions or seek clarification, fostering an environment of engagement and understanding. These strategies were essential for capturing authentic inmate perspectives and ensuring the reliability of our findings.

For prison managers, the practical implications include considerations for the coach selection and training process. The emphasis should be focused on clear communication, adaptability, and the cultivation of balanced interpersonal relationships. Creating a supportive environment is crucial and requires resources for coaches to develop the necessary interpersonal skills and foster a climate of trust and respect. Sensitivity to inmate backgrounds is essential, given the diverse experiences and potential trauma they may have faced. Coaches should be trained to interact with care while avoiding potentially triggering topics.

The balance between an authentic and energetic demeanor, including a certain degree of strictness, emerges as a key factor in effective coaching within the prison context. Prison management should encourage a leadership style that combines passion and inspiration with the authority required for the setting. Acknowledging the significance of coaches' physical appearance in earning respect from inmates suggests a consideration of fitness and sporting abilities in the selection process. In conclusion, the implementation of these practical implications can contribute to the refinement of coaching programs in prisons, fostering positive development and growth of inmates. Furthermore, these insights can guide future research endeavors aimed at continually improving coaching effectiveness within the unique context of prisons.

The study's limitations point to promising directions for future research. First, the exclusive focus on younger men inmates in open prisons might not fully capture the experiences of older prisoners or women, suggesting the need to explore other prison environments for distinct insights. For example, a more restrictive or closed prison where inmates are not allowed to leave for school or work might offer some very different perspectives. Second, the participation of numerous coaches may have introduced variability in the coaching experience, potentially affecting the consistency of the outcomes. Inmates might have responded differently to various coaching styles, which could lead to varied perceptions of effectiveness and impact on their development. This diversity in coaching could be both a strength, in terms of providing comprehensive exposure, and a challenge, in terms of achieving uniformity in results. For further research, it would be useful to investigate the question of whether the effectiveness of the coaching program can be better increased by a uniform approach with a few coaches or by a variety of coaching styles with several coaches. Third, future studies should also consider interviews with coaches, especially with coaches working full-time in the prison context. Given that the coach-athlete relationship develops over time by sharing moments outside of the coaching situations (74), this approach would provide valuable insights into coaching dynamics directly from those persons immersed in the work with inmates over an extended period. The question, how coaches can effectively balance confidence with reflective practice, could also be addressed in this study.

## Data Availability

The datasets presented in this article are not readily available because the participants of this study did not provide written consent for their data to be shared publicly; therefore, so due to the sensitive nature of the research supporting data is not available. Requests to access the datasets should be directed to Milan Dransmann, m.dransmann@uni-bielefeld.de.
